# The psychological well-being and prenatal bonding of gestational surrogates

**DOI:** 10.1093/humrep/dey048

**Published:** 2018-02-23

**Authors:** N Lamba, V Jadva, K Kadam, S Golombok

**Affiliations:** 1 Centre for Family Research, University of Cambridge, Free School Lane, Cambridge, UK; 2 Corion Fertility Clinic, Trans Avenue, Lokhandwala Road, Andheri (West), Mumbai, India

**Keywords:** cross-border, surrogacy, India, depression, prenatal bonding, quantitative research, health policy

## Abstract

**STUDY QUESTION:**

How does the psychological well-being and prenatal bonding of Indian surrogates differ from a comparison group of mothers?

**SUMMARY ANSWER:**

Surrogates had higher levels of depression during pregnancy and post-birth, displayed lower emotional connection with the unborn baby, and greater care towards the healthy growth of the foetus, than the comparison group of mothers.

**WHAT IS ALREADY KNOWN:**

Studies in the West have found that surrogates do not suffer long-term psychological harm. One study has shown that surrogates bond less with the foetus than expectant mothers.

**STUDY, DESIGN, SIZE, DURATION:**

This study uses a prospective, longitudinal and cross-sectional design. Surrogates and a matched group of expectant mothers were seen twice, during 4–9 months of pregnancy and 4–6 months after the birth.

**PARTICIPANTS/MATERIALS, SETTING, METHODS:**

Semi-structured interviews and standardized questionnaires were administered to 50 surrogates and 69 expectant mothers during pregnancy and 45 surrogates and 49 expectant mothers post-birth. All gestational surrogates were hosting pregnancies for international intended parents.

**MAIN RESULTS AND THE ROLE OF CHANCE:**

Surrogates had higher levels of depression compared to the comparison group of mothers, during pregnancy and post-birth (*P* < 0.02). Low social support during pregnancy, hiding surrogacy and criticism from others were found to be predictive of higher depression in surrogates post-birth (*P* < 0.05). Regarding prenatal bonding, surrogates interacted less with and thought less about the foetus but adopted better eating habits and were more likely to avoid unhealthy practices during pregnancy, than expectant mothers (*P* < 0.05). No associations were found between greater prenatal bonding and greater psychological distress during pregnancy or after relinquishment.

**LIMITATIONS, REASONS FOR CAUTION:**

All surrogates were recruited from one clinic in Mumbai, and thus the representativeness of this sample is not known. Also, the possibility of socially desirable responding from surrogates cannot be ruled out.

**WIDER IMPLICATIONS OF THE FINDINGS:**

As this is the first study of the psychological well-being of surrogates in low-income countries, the findings have important policy implications. Providing support and counselling to surrogates, especially during pregnancy, may alleviate some of the psychological problems faced by surrogates.

**STUDY FUNDING/COMPETING INTEREST(S):**

This study was supported by the Wellcome Trust [097857/Z/11/Z] and Nehru Trust, Cambridge. K.K. is the Medical Director of Corion Fertility Clinic. All other authors have no conflict of interest to declare.

## Introduction

Commercial surrogacy in India became a global phenomenon in 2002 (Pande, 2011). Factors such as allowing the intended parents’ names to be on the birth certificate, cutting-edge technology and cheaper medical costs attracted intended parents from around the world towards commercial gestational surrogacy in India (Smerdon, 2008; Karandikar *et al.*, 2014). In gestational surrogacy, a surrogate lacks a genetic link to the child. It is estimated that 25 000 children were born through surrogacy in India up until 2015 (Söderström-Anttila *et al.*, 2016) and terms such as ‘womb farm’ (Moorti, 2011), ‘baby factory’ (Roberts, 2012) and ‘market pregnancy’ (Rudrappa, 2015) were frequently used to represent this burgeoning ‘business’. This unregulated surrogacy market is believed to be worth $2.3 billion (Deonandan *et al.*, 2012). However, media stories about stateless and parentless surrogacy children, and concerns regarding the poor treatment of surrogates, resulted in the government of India drafting a new Surrogacy (Regulation) Bill in 2016 to ban commercial surrogacy, deeming it exploitative. The Bill permits only altruistic surrogacy for infertile Indian couples (Sibal, 2016).

It has been argued that cross-border surrogacy with its legal, political, ethical, religious and procedural challenges puts the well-being of the surrogates, intended parents and unborn children at serious risk (Pennings *et al.*, 2008; Crockin, 2013; Söderström-Anttila *et al.*, 2016). In particular, it has been suggested that large income gaps and extreme power differentials between intended parents and surrogates (Dasgupta and DasGupta, 2014), the commodification of women’s bodies in poverty-stricken populations (Baumhofer, 2012) and a lack of alternative choices for surrogates (Pande, 2009a) made surrogates in India vulnerable to exploitation. Moreover, since women from lower-income populations in developing nations suffer from high levels of emotional problems, primarily prenatal and postnatal depression, compared to women from higher-income nations, surrogacy candidates in India may already be at risk for psychological problems (WHO, 2017). These concerns have led to considerable unease regarding surrogates’ psychological well-being; however, the potential psychological distress experienced by surrogates has not been studied in the Global South (Crockin, 2013; Söderström-Anttila *et al.*, 2016).

Specific factors that may cause psychological harm to surrogates, also remain largely unexplored. Critics of surrogacy argue that women form a deep bond with the unborn baby and that it is emotionally distressing for a woman to give up a child that has been nurtured in her womb (Warnock Report, 1985; British Medical Association, 1996). In non-surrogacy pregnancies, lower maternal–foetal bonding has been found to be associated with decreased positive health practices during pregnancy (Lindgren, 2001). For surrogates, it has been suggested that detaching from the foetus could lead to surrogates putting the unborn child’s health at risk by engaging in risky behaviours such as smoking or not eating well (British Medical Association, 1996; Jadva, 2016).

Only three studies have examined maternal–foetal bonding or attitudes towards the foetus in the context of surrogacy (Fischer and Gillman, 1991; van den Akker, 2007; Lorenceau *et al.*, 2015), all of which were conducted in the USA or Europe. Fischer and Gillman (1991) administered the Maternal Fetal Attachment Scale (Cranley, 1981) to 21 surrogates and 21 expectant mothers in the USA and found that surrogates were significantly less attached to the unborn child than were the non-surrogates. In contrast, in France, Lorenceau *et al.* (2015), found surrogates to show levels of attachment to the foetus that fell within the normal range for non-surrogate pregnant women. However, only 11 surrogates participated in the study and there was no comparison group. van den Akker (2007) found that surrogates were significantly less concerned about the health and well-being of the foetus and less positive about the foetus than were intended mothers.

In terms of relinquishment, research in the USA and the UK has repeatedly shown that most surrogates are able to hand over the baby they carry (Ragoné, 1994; Jadva *et al.*, 2003, 2012; van den Akker, 2003; Imrie and Jadva, 2014). It has been suggested that they make a conscious effort to think of surrogacy as a job and do not see the baby as their own (Snowdon, 1994; Baslington, 2002). Payment in commercial surrogacy is also thought to be a contributing factor in creating an emotional distance between the surrogate and the developing foetus (Baslington, 2002).

However, studies conducted in the West cannot be generalized to the Global South, mainly due to the large cultural differences in the way in which surrogacy is practiced and legislated (Pande, 2009a; Crockin, 2013; Söderström-Anttila *et al.*, 2016). Sociologists and anthropologists, through their in-depth fieldwork, have studied the lived experiences of Indian surrogates (Pande, 2009a, 2010a, 2011; Vora, 2014; Rudrappa, 2015). In India, surrogates are generally recruited by agents via word of mouth. Pande described surrogates as ‘docile, selfless and nurturing’ women who were trained to be perfect ‘worker-mothers’ (Pande, 2010a). She found that Indian surrogates viewed their connection to the foetus as arising through blood ties (shared substance) and sweat (the labour of gestation) rather than the genetic connection that is emphasized in Western countries. It is not known if these feelings of connection to the foetus are similar to the concept of prenatal bonding and if so, whether developing such a bond has consequences for surrogates’ psychological well-being during pregnancy and following the birth.

Surrogates in India often live in a surrogate house, which is group accommodation located near fertility clinics, during their pregnancy. While living in the surrogate house enabled them to be under ‘constant surveillance’ by clinic staff it has also been reported to provide surrogates with a feeling of sisterhood (Pande, 2011; Vora, 2014).

Indian surrogates often receive relatively large sums of money, amounting to ~10 years’ worth of income (Pande, 2009a). Unlike the USA or the UK, surrogates in India are unlikely to meet the intended parents after the birth of the child (Pande, 2011). The clinic, as the mediator between the intended parents and the surrogate, tends to depersonalize the role of the surrogate (Vora, 2014), although the surrogates themselves often hope for a lasting bond and act of reciprocity and generosity from the intended parents (Pande, 2011). Other complications such as language barriers and distance may make direct relationships between the surrogates and intended parents difficult.

Surrogacy in India, unlike the West, is frequently kept a secret by the surrogate and her family as it is considered immoral (Pande, 2009b). Surrogates face social humiliation and criticism from family members and the wider community, and may be shunned by persons in these networks (Karandikar *et al.*, 2014). In particular, it is common for uneducated or misinformed family or friends to consider pregnancy outside the realm of marriage as sex-work or adultery, thus leading to ‘sexualized stigmatization’ (Deonandan *et al.*, 2012; Pande, 2009b). These experiences may negatively affect surrogates’ psychological well-being. There is a large body of research showing that social stigma is likely to generate feelings of depression and anxiety (Markowitz, 1998; Schmitt *et al.*, 2014). For those who decide to hide their surrogacy, the visible baby bump makes it impossible for them to meet family and friends during the pregnancy, resulting in social isolation and a lack of social support. Moreover, unlike western countries, not only is the detailed screening for psychopathology in surrogates typically omitted, psychological counselling and support are also not readily available to surrogates in India, potentially making them more vulnerable to psychological problems (Palattiyil *et al.*, 2010; Karandikar *et al.*, 2014).

The aim of the present study was to examine the psychological well-being of Indian surrogates and the nature of their prenatal bond to the baby. A further aim was to assess the association between surrogates’ experiences and their psychological well-being.

## Materials and Methods

### Design and participants

Fifty Indian surrogates were compared with a demographically matched group of 69 expectant mothers. The women were assessed at two time points: (i) Phase 1: during the fourth−ninth month of pregnancy and (ii) Phase 2: 4–6 months after the birth of the baby.

In line with the guidelines from the Indian Council of Medical Research (ICMR), all surrogates were gestational surrogates, had at least one child of their own from a current or previous marriage, and were screened for their physical and mental health. Surrogates were recruited by an agency working with Corion Fertility Clinic, Mumbai, over a two-year period. Surrogates who were in the second or third trimester of pregnancy were invited to take part by the clinic administrator. All agreed to participate, yielding a response rate of 100%. Approximately 4–6 months after the birth of the baby, the clinic administrator contacted the surrogates again for the follow up interview. Overall, 45 surrogates agreed to take part, representing a response rate of 90%. All surrogates were hosting pregnancies for international intended parents.

The comparison group of expectant mothers was recruited from four public hospitals in Mumbai and Delhi. Expectant mothers were recruited by the interviewer (N.L.) from hospital waiting rooms. The interviews were conducted at the hospitals. The expectant mothers were matched as closely as possible to the surrogates according to age, educational level, socio-economic background and month of pregnancy. In addition, they had to have at least one child. Of the 78 expectant mothers who fulfilled the inclusion criteria, 88% agreed to take part in the study. For Phase 2, these mothers were contacted directly by the researcher. Of the 69 mothers who took part in Phase 1, 49 mothers took part in Phase 2, representing a participation rate of 71%.

The interviews were conducted in a private room in Hindi by N.L., a native Hindi speaker. Written or verbal (recorded) consent was obtained before starting the interview. Each participant received ₹1000 (£12) for taking part in each interview. N.L. translated and transcribed the interviews from Hindi to English for analysis. Ethical approval for the study was obtained from the University of Cambridge Psychology Research Ethics Committee and the Corion Fertility Clinic’s Ethics Committee.

### Measures

Standardized questionnaires and face-to-face interviews were administered to all participants. The questionnaires were read out by the interviewer as the majority of the participants could not read or write.

#### Psychological well-being

Participants were administered the Anxiety, Depression and Stress Scale (ADSS) (Bhatnagar, 2011) at Phase 1 and Phase 2 of the study. The ADSS was developed in India and the standardization included participants from illiterate and marginalized groups. The 48-item scale comprises three subscales: anxiety (19 items), depression (15 items) and stress (14 items), with higher scores indicating greater psychological problems. The percentile cut-off for ‘severe’ anxiety, depression and stress was P_76_–P_100_. Participants responded ‘yes’ or ‘no’ to questions such as ‘I feel more nervous and anxious than usual’ and ‘Often I want to be alone’. The internal consistency of the original scale was 0.81. Cronbach alphas for the current sample for each subscale in both phases of the study were >0.85.

#### Prenatal bonding

An adaptation of the Maternal Fetal Attachment Scale (MFAS) (Cranley, 1981) was administered to assess the extent to which pregnant women had bonded with the unborn baby. The original scale comprised 24-item rated on a 5-point Likert scale. The current study used a modified version of the MFAS with yes/no response options to aid understanding by the participants. Higher scores represented greater bonding. Sample items included: ‘I talk to the unborn baby’ and ‘I do things to try to stay healthy that I would not do if I were not pregnant’.

A principal component analysis using varimax rotation was conducted on the modified MFAS. Items with eigen values below 0.4 (5 items) and items with negative loadings (1 item) were excluded. A modified scale was produced which assessed feelings, thoughts and actions towards the foetus during pregnancy. Two factors were identified: (i) Emotional Prenatal Bonding and (ii) Iinstrumental Prenatal Bonding, with 10 and 5 items, respectively (Table I). The Emotional Prenatal Bonding scale measured the level of interaction women had with the foetus and whether they had attributed characteristics to the foetus. The Instrumental Prenatal Bonding scale assessed the extent to which women were attentive and caring towards the foetus. Cronbach *α* were 0.74 and 0.59 for Emotional Prenatal Bonding and Instrumental Prenatal Bonding, respectively. The factor scores for the two subscales were used in further analyses.

**Table I:** Factorial structure of maternal–foetal attachment subscales.

Item	Factor loadings	
	Factor 1	Factor 2
I can hardly wait to hold the baby.	0.625	
I can almost guess what the baby’s personality will be from the way he/she moved around.	0.615	
It seems the baby kicks and moves to tell me it’s eating time.	0.604	
I wonder if the baby thinks and feels ‘things’ inside of me.	0.578	
I poke the baby to get him/her to poke back.	0.535	
I talk to the unborn baby.	0.506	
I wonder if the baby can hear inside of me.	0.500	
I imagine myself taking care of the baby.	0.477	
I decided on a name for a baby boy	0.436	
I refer to the baby by a nickname	0.408	
I give up doing certain things because I want to help the baby.		0.702
I eat meat and vegetables to be sure the baby gets a good diet.		0.701
I do things to try to stay healthy that I would not do if I were not pregnant.		0.502
I stroke my tummy to quiet the baby when there is too much kicking.		0.497
I try to picture what the baby will look like.		0.482

*Note*: Rotated component matrix for the attachment scale; extraction method was principal component analysis, and rotation method was Varimax; only factor loadings over 0.40 are shown. Hindi version utilized in the study amended the items from ‘my baby’ to ‘the baby’ for all items.

#### Experiences of surrogacy

Semi-structured interviews were conducted with the surrogates to obtain detailed information on their experiences of surrogacy. A flexible style of questioning was used to collect information and the responses were coded according to a standardized coding scheme. At Phase 1 the following variables were coded: (i) *Hiding surrogacy*: ‘from everyone’, ‘from most people’ or ‘did not hide surrogacy’; (ii) *Criticism for being a surrogate*: ‘much criticism’ or ‘little or no criticism’; (iii) *Feelings about living in the surrogate house*: ‘positive’, ‘neutral’ or ‘negative’; (iv) *Perceived support during pregnancy*: ‘sufficient support’, or ‘no support’; and (v) *Satisfaction with payment*: ‘satisfied’, ‘somewhat satisfied’ or ‘not satisfied. At Phase 2, data were obtained on: (vi) *Meeting the newborn*: ‘yes’ or ‘no’ and (vii) *Meeting intended parents*: ‘yes’ or ‘no’.

### Statistical analyses

A series of 2 × 2 mixed ANOVAs, with group (surrogates versus expectant mothers) and time (during pregnancy versus after the birth) as factors, were conducted to examine differences between groups over time, separately for anxiety, depression and stress. A MANOVA was conducted to examine differences between the surrogates and non-surrogates during pregnancy for the two subscales of the revised MFAS. Correlations and multiple regression analyses were used to examine factors associated with surrogates’ psychological well-being during pregnancy and following the birth, and with the prenatal bonding subscales. Where a significant correlation was found between a demographic variable and a dependent variable, the analysis was conducted with the demographic variable included as a covariate.

## Results

### Characteristics

The characteristics of the sample are shown in Table II. There was no difference between groups in the age of the mothers, *F*(1, 118) = 3.35, *P* = 0.07, income level, *F*(1, 110) = 0.49, *P* = 0.48, or religious affiliation, *χ*^2^(1) = 1.01, *P* = 0.31. However, surrogates had more children, *χ*^2^(1) = 21.73, *P* < 0.01, were more likely to be single (widowed, abandoned or divorced), *χ*^2^(1) = 23.92, *P* < 0.01, were less educated *χ*^2^(2) = 13.72, *P* < 0.01, and were interviewed earlier in their pregnancy *F*(1, 118) = 86.46, *P* < 0.01, compared to expectant mothers. In total, 82% of surrogates had been employed before becoming a surrogate. The majority had worked as domestic helpers (61%) or as semi-skilled labourers in shops, factories or at home (24%).

**Table II:** Sample characteristics.

	Surrogates		Expectant mothers			
	Mean	SD	Mean	SD	*F*	*P*
Age (years)	27.6	2.51	26.6	3.46	3.35	0.07
Month of pregnancy	6.2	1.18	8.3	1.14	86.46	0.00
Monthly income (INR.)	8042	4005	7593	2718	0.49	0.48
	***n*(%)**		***n*(%)**		***χ*^2^**	***P***
Number of children					21.73	0.00
1	18 (36)		55 (80)			
2 or more	32 (64)		14 (20)			
Religion					1.01	0.31
Hindu	24 (48)		43 (62)			
Muslim	23 (46)		27 (38)			
Other	3 (6)		0 (0)			
Educational status					13.72	0.00
No schooling	23 (44)		10 (15)			
First−seventh grade	10 (20)		18 (26)			
7th−12th grade	17 (34)		41 (59)			
Marital status					23.92	0.00
Husband	33 (66)		68 (99)			
No husband	17 (34)		1 (1)			

*Note*: For ‘religion’, codes were collapsed to ‘Hindu and Muslim’ for Chi square analyses.

### Psychological well-being

For depression, a 2×2 mixed ANOVA found a significant main effect for group, *F*(1, 85) = 6.50, *P* = 0.01, indicating higher levels of depression among surrogates than the comparison group of mothers. There was no significant effect for time and no significant interaction between group and time, showing that the surrogates had higher levels of depression than expectant mothers during pregnancy and after the birth of the baby. During pregnancy, 36% of surrogates obtained scores above the cut-off point for severe depression compared with 13.8% of expectant mothers (*χ*^2^(1) = 12.9, *P* < 0.001). Following the birth, the percentages of surrogates and expectant mothers who scored above the cut-off point for severe depression were 27.3 and 13.3%, respectively, (*χ*^2^(1) = 6.12, *P* = 0.01).

For anxiety and stress, separate 2×2 mixed ANOVAs found no significant main effects or interaction effects, showing that there were no significant differences between surrogates and expectant mothers in either anxiety or stress during pregnancy or after the birth of the baby (Table III).

**Table III:** 2 × 2 Mixed ANOVA for psychological well-being scores.

Variables	Surrogates		Expectant mothers		Surrogates versus expectant mothers		Pre versus post pregnancy		Interaction between group×time	
	Mean	SD	Mean	SD	*F*	*P*	*F*	*P*	*F*	*P*
Anxiety during pregnancy	9.6	5.50	7.7	4.79	0.95	0.33	1.14	0.28	1.22	0.27
Anxiety post-birth	8.2	5.39	7.7	5.26						
Depression during pregnancy	8.4	4.88	6.1	4.06	6.50	**0.01**	2.31	0.13	0.02	0.86
Depression post-birth	7.6	4.88	5.4	4.47						
Stress during pregnancy	7.8	4.69	6.8	4.07	1.2	0.27	0.06	0.80	0.00	0.98
Stress post-birth	7.7	4.70	6.7	4.22						

### Maternal–foetal bonding

The two subscales were entered into a MANOVA. Wilks’s *λ* was significant, *F*(2, 116) = 4.40, *P* = 0.01. One-way ANOVAs showed a significant difference between surrogates and expectant mothers in Emotional Prenatal Bonding, *F*(1, 116) = 4.19, *P* = 0.04, with surrogates showing lower emotional connection with the foetus than expectant mothers. There was also a significant difference between surrogates and expectant mothers for Instrumental Prenatal Bonding, *F*(1, 116) = 4.27, *P* = 0.04, reflecting greater care and attention towards the foetus by surrogates than the expectant mothers (Table IV).

**Table IV:** Group differences in maternal–foetal bonding subscales scores.

Variables	Surrogates		Expectant mothers			
	Mean	SD	Mean	SD	*F*	*P*
Factor 1: Emotional Prenatal Bonding	−0.21	1.23	0.16	0.75	4.23	**0.04**
Factor 2: Instrumental Prenatal Bonding	0.21	0.68	−0.16	1.16	4.19	**0.04**

### Experiences of surrogacy

#### Phase 1: During pregnancy

All of the surrogates were hiding their involvement in surrogacy to some extent. Overall, 32% of surrogates (*n* = 16) were hiding surrogacy from everyone and 68% of surrogates (*n* = 34) were hiding surrogacy from most people. With respect to criticism for being a surrogate, 26% (*n* = 13) of surrogates reported experiencing criticism. In total, 74% of the surrogates (*n* = 37) reported feeling positive about living in the surrogate house, with the remaining 26% (*n* = 13) reporting neutral rather than negative feelings. Regarding support, 66% (*n* = 33) of surrogates felt that they had received sufficient support during pregnancy. And 34% (*n* = 17) of the surrogates felt unsupported. In terms of payment, the majority of the surrogates (74%, *n* = 37) reported being satisfied with the amount of payment they were receiving, 18% (*n* = 9) were somewhat satisfied, and 8% (*n* = 4) said they were not satisfied with the amount.

#### Phase 2: After the birth

All surrogates reported a desire to meet the intended parents and the baby. Two-thirds of the surrogates (64%, *n* = 32) did not see the baby and just over half of the surrogates (54%, *n* = 24) did not meet the intended parents following the birth. These surrogates expressed uncertainty about whether or not they would ever be allowed to meet the intended parents and the baby. When surrogates had met the intended parents following the birth, the meetings were usually brief, ranging from 5 to 20 min, and were supervised by a member of staff from the clinic who translated their conversations.

### Factors associated with surrogates’ psychological well-being

Based on the previous literature, the following variables relating to surrogates’ characteristics and experiences were correlated with their prenatal and postnatal depression scores: demographic factors (socio-economic status, educational status and marital status), pregnancy (support during pregnancy), bonding with the foetus (Emotional Prenatal Bonding and Instrumental Prenatal Bonding), surrogacy arrangement (satisfaction with payment and feelings towards surrogate house) and stigmatization (hiding surrogacy and facing criticism). Factors that were unique to the second phase of the study (meeting the newborn and meeting the intended parents after delivery) were correlated with depression scores at Phase 2. None of the variables was significantly correlated with depression during pregnancy. However, support during pregnancy (*r* = −0.41, *P* = 0.00), hiding surrogacy (*r* = 0.30, *P* = 0.04) and criticism (*r* = 0.33, *P* = 0.02), were significantly correlated with postnatal depression scores. The combined effect of these variables was examined using a multiple linear regression. It was found that lower perceived support during pregnancy, hiding surrogacy and facing criticism for being a surrogate significantly predicted higher depression in surrogates after the birth of the baby, *F*(3, 43) = 8.36, *P* < 0.001, adjusted *R*^2^ = 0.38, accounting for 38% of the variance in depression after the birth. All three values added significantly to the prediction, *P* < 0.05 (Table V).

**Table V:** Factors predicting higher depression in surrogates.

Variables	Predictors	*β*	*t*	*P*	Adjusted *R*^2^
Depression (post-birth)					0.32
	Perceived support during pregnancy	−0.38	−3.02	0.00	
	Hiding surrogacy	0.37	2.94	0.00	
	Facing criticism	0.31	2.44	**0.02**	

### Factors associated with surrogates’ bonding to the unborn baby

The variables relating to surrogates’ characteristics and experiences and their psychological well-being (i.e. anxiety, depression, and stress scores during pregnancy and following the birth) were correlated with the MFAS subscales. Although there were no significant correlations with Instrumental Prenatal Bonding, Emotional Prenatal Bonding was found to be correlated with educational status (*r *= −0.31, *P* = 0.03) and feelings towards the surrogate house (*r* = 0.35, *P* = 0.01), with surrogates of lower educational status and who had a positive experience at the surrogate house being more likely to emotionally bond with the foetus. In order to examine the combined effects of educational status and feelings towards the surrogate house, both variables were entered into a hierarchical multiple regression. It was found that after controlling for educational status, surrogates’ feelings towards the surrogate house still significantly predicted their emotional connection with the foetus, *F*(2, 45) = 4.82, *P* = 0.01, *R*^2^ = 0.14, accounting for 14% of the variance in their emotional prenatal bonding (Table VI). There were no significant correlations between surrogates’ prenatal bonding and psychological well-being scores.

**Table VI:** Factors predicting surrogates’ emotional prenatal bonding.

Variables	Predictors	*β*	*t*	*P*	Adjusted *R*^2^
Emotional Prenatal Bonding					0.14
	Educational status	−0.23	−1.65	0.10	
	Feeling towards the surrogate house	0.29	2.05	**0.04**	

## Discussion

The findings of the study showed that Indian surrogates experienced higher levels of depression across pregnancy and several months following the birth, than the comparison group of mothers who were having their own babies. However, there was no difference in levels of depression from the time of pregnancy to after the birth for either group, indicating that giving up the newborn did not add to surrogates’ levels of depression. Instead, it appears that the surrogates were more depressed from pregnancy onwards and may have been more depressed before they initiated the surrogacy arrangement. There were no differences between the surrogates and the expectant mothers for anxiety and stress.

An examination of factors associated with depression in surrogates following relinquishment of the baby found that lack of support during pregnancy, hiding surrogacy from family and friends, and being criticized by family or neighbours for becoming a surrogate were each found to be risk factors for depression. These factors reflect the stigmatization associated with surrogacy in India. Most of the surrogates had kept their involvement in surrogacy secret from their family and community due to anticipated stigma. As secrecy and experiencing stigma can have a negative impact on psychological well-being (Kelly, 2002; Schmitt *et al.*, 2014), this may have played a part in the raised levels of depression shown by surrogates. The majority of the surrogates reported feeling positive about living in the surrogate house. Staying there may have made them feel more supported and less burdened by the pressures of maintaining a secret from those around them and free of stress of everyday life. Additionally, surrogates’ satisfaction with the payment they received for surrogacy did not facilitate their psychological well-being.

In terms of bonding with the unborn baby, surrogates bonded emotionally with the foetus less than expectant mothers; they were less likely to think about and interact with the foetus. This finding is in line with the only comparable study in the field, which found that surrogates bonded less with the unborn child than did expectant mothers (Fischer and Gillman, 1991). It has been suggested that viewing surrogacy as paid employment may help surrogates keep an emotional distance from the foetus (Snowdon, 1994; Baslington, 2002). The intention of not being a parent and the anticipated separation after the birth may also facilitate the emotional distance from the unborn baby (Braverman *et al.*, *et al.*, 2012; Jadva, 2016).

However, surrogates exhibited higher levels of instrumental bonding with the unborn baby than the expectant mothers. This may be reflective of how pragmatically surrogates abided by their contract and commitment to deliver a healthy baby to the intended parents. Whilst it is not surprising that surrogates allotted time and effort to nurture and protect the foetus, it was unexpected that they did so more than women carrying their own babies. This may also result from structural and cultural aspects of Indian surrogacy where the daily life of an Indian surrogate living in a surrogacy house entails one purpose, that is, to care for the foetus and deliver it at full term. All of her activities are structured around being a responsible surrogate. These findings are in accordance with Pande’s (2010a) concept of ‘worker-mother’ duality, whereby surrogates limit their role as a ‘mother’ by not connecting emotionally with the foetus but responsibly follow their role as a ‘worker’ by being vigilant towards the needs of the foetus.

In examining factors associated with prenatal bonding, it was found that surrogates who had a more positive experience at the surrogate house were more likely to be emotionally involved with the foetus. Surrogates who were happy at the surrogate house may have felt more immersed in their role as a surrogate. It was further observed that surrogates with no education displayed higher emotional involvement with the unborn baby, than did those with at least some education. This finding suggests that lack of education may interfere with surrogates’ ability to regulate their feelings towards the unborn child. This could be concerning as surrogates are generally expected to keep an emotional distance from the foetus (Baslington, 2002).

Contrary to expectations, greater bonding with the unborn baby was not found to be associated with raised levels of depression in the surrogates during pregnancy and post-birth. This challenges the widely held assumption that surrogates who develop strong bonds to the unborn baby would show higher levels of psychological problems.

Similar to findings in the West (Ragoné, 1994; Jadva *et al.*, 2003; van den Akker, 2003; Imrie and Jadva, 2014), Indian surrogates in the present study were able to relinquish the newborn. It is noteworthy, however, that the majority of surrogates did not meet the baby to whom they had given birth nor did they meet the intended parents. This contrasts sharply with the practice of surrogacy in the West where some surrogates remain in contact with the family they have helped create as the child grows up (Jadva *et al.*, 2015; Blake *et al.*, 2016). Also, not being able to see, hold and meet the newborn, which is viewed as an important aspect of a successful surrogacy arrangement (Hohman and Hagan, 2001), may have led to dissatisfaction about the surrogacy. A qualitative analysis of surrogates’ feelings about not seeing the baby or the intended parents is described elsewhere (Lamba and Jadva, 2018). Living in uncertainty about whether or not they would meet with the baby and the intended parents, even a few months after the birth, appeared to be psychologically stressful for the surrogates.

A limitation of the study was that all of the surrogates were recruited from one clinic in Mumbai, and thus the findings may not be representative of surrogates’ experiences at different clinics in India. Conducting interviews at the clinic may have led to socially desirable responding, as surrogates may have been afraid of the negative consequences of expressing any disagreements with clinic staff over accommodation, payment or medical assistance. However, given that most had not disclosed their decision of becoming a surrogate to most of their family members, it would have been unethical to contact them and interview them in their home environments. Surrogates were informed at the beginning of the study that no information would be shared with the clinic. Another disadvantage is that, unlike surrogates, expectant mothers may have had an unplanned pregnancy. Controlling for a planned pregnancy may have resulted in even greater differences in depression between the two groups as unplanned pregnancy is associated with raised levels of depression. A further limitation relates to the low internal reliability of the instrumental bonding subscale utilized in the present study. Additionally, there was a lower response rate (71%) for the expectant mothers than for the surrogates during the second phase of the study, which may have introduced a bias in terms of maternal depression. However, a lower response rate was not surprising as they had a newborn to attend to at home. Finally, this study lacks information on the history of participants’ mental health prior to pregnancy.

An advantage of the study is that it is the only investigation to have followed up surrogates hosting pregnancies for international intended parents in India from the time of pregnancy until several months after relinquishment of the baby. It is also the only study to have examined the psychological health and feelings towards the foetus of surrogates in the Global South. Importantly, the study examined risk factors for psychological problems, which makes the findings more meaningful in terms of understanding which aspects of surrogacy may impact surrogates’ mental health.

While the present study was conducted prior to the new Surrogacy Bill in India, most of the countries in the Global South are going through a transient phase regarding their laws on surrogacy, therefore, the findings have important policy implications. Since the present Indian Bill stipulates that the surrogate should be a close family relative, there is a fear that this change in the law may result in surrogacy arrangements becoming secret family affairs. This may perpetuate stigma against surrogacy in Indian society and increase the risk of psychological harm to surrogates. Informing practitioners and clinics about the importance of support during pregnancy and offering counselling to surrogates to help tackle the burden of social stigma and social disapproval, may alleviate some of the psychological issues faced by surrogates. More detailed psychological screening of surrogates prior to entering into surrogacy is also highly recommended.

While the new policy proposed by the Indian government may ensure that surrogates receive support from family members and meet the baby after the birth, it has its limitations. For instance, within a family, issues concerning failed pregnancies, multiple abortions, and miscarriages may lead to more blame and guilt. Also, whilst the surrogates in the present study lived in uncertainty regarding their meeting with the newborn and intended parents, the new Bill suggests that the surrogate—being a close family relative—may feel forced to maintain a relationship with these parties (perhaps in close proximity) for the long-term. Thus, the surrogate still may not get to choose the level of involvement she has with the intended parents and the child.

## References

[dey048C1] Baslington H The social organization of surrogacy: relinquishing a baby and the role of payment in the psychological detachment process. J Health Psychol 2002;7:57–71. 22114227 10.1177/1359105302007001652

[dey048C2] Baumhofer E *Commodifying the Female Bod*y: Outsourcing Surrogacy in a Global Market. California: UCLA Center for the Study of Women, 2012.

[dey048C3] Bhatnagar P *Anxiety, Depression and Stress Scale* National Psychological Corporation. 2011.

[dey048C4] Blake L, Carone N, Slutsky J, Raffanello E, Ehrhardt AA, Golombok S Gay father surrogacy families: relationships with surrogates and egg donors and parental disclosure of children’s origins. Fertil Steril 2016;6:1503–1509. 10.1016/j.fertnstert.2016.08.013 PMC5090043 27565261

[dey048C5] Braverman A, Casey P, Jadva V Reproduction through surrogacy: The UK and USA experience In: Richards M, Pennings G, Appleby JB (eds) Reproductive Donation: Policy, Practice and Bioethics. Cambridge, UK: Cambridge University Press, 2012.

[dey048C6] British Medical Association Changing conceptions of motherhood In: The Practice of Surrogacy in Britain. London: British Medical Association, 1996.

[dey048C7] Cranley MS Development of a tool for the measurement of maternal attachment during pregnancy. Nurs Res 1981;30:281–284. 6912989

[dey048C8] Crockin SL Growing families in a shrinking world: legal and ethical challenges in cross-border surrogacy. Reprod Biomed Online 2013;27:733–741. 24120561 10.1016/j.rbmo.2013.06.006

[dey048C9] Dasgupta S, DasGupta S Globalization and Transnational Surrogacy in India: Outsourcing Life. UK: Lexington Books, 2014.

[dey048C10] Deonandan R, Green S, van Beinum A Ethical concerns for maternal surrogacy and reproductive tourism. J Med Ethics 2012;12:742–745. 10.1136/medethics-2012-100551 23047836

[dey048C11] Fischer S, Gillman I Surrogate motherhood: attachment, attitudes and social support. Psychiatry 1991;54:13–20. 11654012

[dey048C12] Hohman MM, Hagan CB Satisfaction with surrogate mothering: a relational model. J Hum Behav Soc Environ 2001;1:61–84.

[dey048C13] Imrie S, Jadva V The long-term experiences of surrogates: relationships and contact with surrogacy families in genetic and gestational surrogacy arrangements. Reprod Biomed Online 2014;29:424–435. 25131555 10.1016/j.rbmo.2014.06.004

[dey048C14] Jadva V Golombok S, Scott R, Appleby JB, Richards M, Wilkinson S Surrogacy: issues, concerns and complexities In: Regulating Reproductive Donation. Cambridge, UK: Cambridge University Press, 2016.

[dey048C15] Jadva V, Blake L, Casey P, Golombok S Surrogacy families 10 years on: relationship with the surrogate, decisions over disclosure and children’s understanding of their surrogacy origins. Hum Reprod 2012;27:3008–3014. 22814484 10.1093/humrep/des273 PMC3442632

[dey048C16] Jadva V, Imrie S, Golombok S Surrogate mothers 10 years on: a longitudinal study of psychological well-being and relationships with the parents and child. Hum Reprod 2015;30:373–379. 25527614 10.1093/humrep/deu339

[dey048C17] Jadva V, Murray C, Lycett E, Maccallum F, Golombok S Surrogacy : the experiences of surrogate mothers. Hum Reprod 2003;18:2196–2204. 14507844 10.1093/humrep/deg397

[dey048C18] Karandikar S, Gezinski LB, Carter JR, Kaloga M Economic necessity or noble cause? A qualitative study exploring motivations for gestational surrogacy in Gujarat, India. J Women Soc Work. 2014.

[dey048C19] Kelly A The Psychology of Secrets. New York: Springer Science & Business Media, 2002.

[dey048C20] Lamba N, Jadva V Psychological Well-being and Prenatal Bonding of Indian Surrogates. London: Palgrave MacMillan, 2018. In press. 10.1093/humrep/dey048 PMC5989605 29566176

[dey048C1000] Lindgren K Relationships among maternal-fetal attachment, prenatal depression, and health practices in pregnancy. Res Nurs Health 2001;24:203–217. 11526619 10.1002/nur.1023

[dey048C21] Lorenceau ES, Mazzucca L, Tisseron S, Pizitz TD A cross-cultural study on surrogate mother’s empathy and maternal–foetal attachment. Women Birth 2015;28:154–159. 25487002 10.1016/j.wombi.2014.11.006

[dey048C22] Markowitz FE The effects of stigma on the psychological well-being and life satisfaction of persons with mental illness. J Health Soc Behav 1998:335–347. 9919855

[dey048C23] Moorti S ‘Womb Farms’ in India: *Orientalism in Scientific Garb* 2011.

[dey048C24] Palattiyil G, Blyth E, Sidhva D, Balakrishnan G Globalization and cross-border reproductive services: ethical implications of surrogacy in India for social work. Int Soc Work 2010;5:686–700.

[dey048C25] Pande A It may be her eggs, but it’s my blood: surrogates and everyday forms of kinship in India. Qual Sociol 2009a;32:379–397.

[dey048C26] Pande A Not an ‘angel,’ not a ‘whore.’ Indian J Gend Stud 2009b;16:141–173.

[dey048C27] Pande A Commercial surrogacy in India: manufacturing the perfect mother-worker. Signs Women Cult Soc 2010a;35:969–992.

[dey048C28] Pande A Transnational commercial surrogacy in India : gifts for global sisters ? Reprod Biomed Online 2011;23:618–625. 21958916 10.1016/j.rbmo.2011.07.007

[dey048C29] Pennings G, de Wert G, Shenfield F, Cohen J, Tarlatzis B Devroey P Devroey. ESHRE Task Force on Ethics and Law 15: cross-border reproductive care. Hum Reprod 2008;23:2182–2184. 18611918 10.1093/humrep/den184

[dey048C30] Ragoné H Surrogate Motherhood: Conception in the Heart. Boulder, CO, and Oxford: Westview Press/Basic Books, 1994.

[dey048C31] Roberts EF God’s Laboratory: Assisted Reproduction in the Andes. California: University of California Press, 2012.

[dey048C32] Rudrappa S Discounted Life: The Price of Global Surrogacy in India. New York: NYU Press, 2015.

[dey048C33] Schmitt MT, Branscombe NR, Postmes T, Garcia A The consequences of perceived discrimination for psychological well-being: a meta-analytic review. Psychol Bull 2014;4:921. 10.1037/a0035754 24547896

[dey048C34] Sibal K Unequal by law. *The Indian Express* [Internet]. 2016 http://indianexpress.com/article/opinion/columns/nda-government-commercial-surrogacy-regulation-bill-reproductive-technologies-3026339/

[dey048C35] Smerdon UR Crossing bodies, crossing borders: international surrogacy between the United States and India. Cumberland Law Rev 2008;39:15.

[dey048C36] Snowdon C What makes a mother? Interviews with women involved in egg donation and surrogacy. Birth 1994;2:77–84. 7857451

[dey048C37] Söderström-Anttila V, Wennerholm UB, Loft A, Pinborg A, Aittomäki K, Romundstad LB, Bergh C Surrogacy: outcomes for surrogate mothers, children and the resulting families-a systematic review. Hum Reprod Update 2016;22:260–276. 26454266 10.1093/humupd/dmv046

[dey048C38] van den Akker O Genetic and gestational surrogate mothers’ experience of surrogacy. J Reprod Infant Psychol 2003;21:145–161.

[dey048C39] van den Akker O Psychological aspects of surrogate motherhood. Hum Reprod 2007;13:53–62. 10.1093/humupd/dml039 16936307

[dey048C40] Vora K Experimental sociality and gestational surrogacy in the Indian ART clinic. Ethnos 2014;79:63–83.

[dey048C41] Warnock M A question of life: report of the committee of inquiry into human fertilisation and embryology. Ir Nurs News 1985;3:7–8. 3846590

[dey048C42] World Health Organisation Mental health: Maternal mental health. 2017.

